# Population growth, accessibility spillovers and persistent borders: Historical growth in West-European municipalities

**DOI:** 10.1016/j.jtrangeo.2017.05.008

**Published:** 2017-06

**Authors:** Chris Jacobs-Crisioni, Eric Koomen

**Affiliations:** a European Commission,^1^1 The views expressed are purely those of the author and may not in any circumstances be regarded as stating an official position of the European Commission. Joint Research Centre, Directorate for Growth and Innovation, Territorial Development Unit, Via E. Fermi 2749, 21027 Ispra, Italy; b Faculty of Economics and Business Administration, VU University Amsterdam, Boelelaan 1105, 1081HV Amsterdam, The Netherlands

## Abstract

Lack of cross-border transport supply has repeatedly been blamed for the fact that national borders limit spatial interaction and, consequently, the growth of border regions. This study applies an accessibility approach to investigate for most municipalities in ten countries in mainland West Europe if foreign transport supply is lagging behind, and if population growth in these municipalities has been affected by the limits that national borders have imposed on market access. To do so, data describing historical population changes and road networks between 1961 and 2011 have been used. The results show that in the study area, cross-border transport accessibility was not at a disadvantage in 1961 and has since then grown even more than domestic accessibility. However, municipal population growth has depended almost exclusively on domestic market access. Processes of economic international integration in the study area are found to coincide with the growth of cross-border accessibility, but do not have a clear coincidence with the effects of cross-border accessibility on population growth.

## 1 Introduction

Potential accessibility, combining transport cost and activity dimensions, is known to be an important determinant of the spatial distribution of economic growth and urban development (Batty, 2013, Hansen, 1959, Keeble et al., 1982, Koopmans et al., 2012, Levinson, 2008, Vickerman et al., 1999). Through cross-border transport links, foreign interaction opportunities spill over national borders and can be an important part of total potential accessibility levels in countries (Stępniak and Rosik, 2013). However, those borders also act as a barrier to interaction (Anderson and Van Wincoop, 2003, Feenstra, 2003), and thus reduce the economic benefits of international accessibility spill-overs. As such, national borders are the cause of inefficiencies in international markets that no doubt affect the spatial distribution of economic and population growth. The European Union (EU) is steering a process of international economic integration in Europe that started in the 1950s (McCormick, 1999, Rodrik, 2000). It may be expected that in the case of complete international economic integration, cross-border interaction opportunities contribute as much to local economic growth as domestic interaction opportunities. Nevertheless, despite the ongoing economic integration efforts, border regions in Europe are often lagging behind in terms of economic growth and population development. The barrier effect that borders still impose is likely a key factor in that lagged growth (Brakman et al., 2012, Redding and Sturm, 2008). Cross-border transport supply is often noted as a key element of border effects. Rietveld (2001) argues that due to the lack of demand for cross-border transportation, the supply of cross-border transport infrastructure lags behind, which again affects foreign market access. Related to this is the idea that transport supply for cross-border interactions is smaller than transport supply for domestic interactions, so that investments in cross-border transportation may improve foreign market access and reduce the impact of border effects (Brülhart, 2011).

To encourage international economic integration in the EU, increase the competitiveness of the EU internal market and reduce economic inequalities, the EU encourages the improvement of cross-border transport links (López et al., 2009) with European Cohesion policies, and transport policies that emerged in the 1990s (Peters, 2003). Recent evidence shows that EU policies have most likely contributed to improvements in accessibility in particular in lagging regions (Ferrara et al., 2016). However, whether European transport policies have reduced local market inefficiencies is debatable. One reason is that unified methods to assess the transnational value of infrastructure projects are unavailable and it is unsure if the supported projects incite market integration (Gutiérrez et al., 2011, van Exel et al., 2002). Another reason is that it is unclear whether the lack of cross-border integration of for instance labour and housing markets is truly caused by lack of transport supply. To explain why cross-border market interaction lags behind, many scholars blame factors that are much harder to change by European policymakers, such as institutional quality (De Groot et al., 2004); cultural and linguistic differences (Chen, 2004, Nitsch, 2000) and the associated higher search costs and lower levels of trust (Linders, 2006); and difficulties with contract enforcement (Rodrik, 2000). In fact, a recent analysis of Belgian commuting flows shows that even without any institutional barriers to regional market integration, cultural and linguistic differences do coincide with considerable regional barriers observable in Belgian labour markets (Persyn and Torfs, 2015).

All in all it is unclear whether improving cross-border connections may improve local cross-border market integration. This study will therefore investigate the effect of changing cross-border interaction opportunities on local population growth levels in the last 50 years in mainland Western Europe. Following similar work of others (Brakman et al., 2012, Koopmans et al., 2012, Redding and Sturm, 2008) we assume that population proxies economic local activity and we resort to this indicator, as a suitably detailed dataset on economic activity is unavailable. The questions that this research will investigate are: is cross-border transport infrastructure supply lagging behind domestic transport supply? Has ongoing international economic integration within the EU coincided with improvements in cross-border accessibility? To what degree have national borders limited market access and thus limited population growth? And did EU economic integration coincide with the impact of cross-border accessibility spillovers on population growth? To do so, we present an analysis of historical domestic and cross-border accessibility trends and a further analysis in which panel data regression methods are used to explore the effect of cross-border interaction opportunities on historical population growth.

The present study is centred on accessibility measures and explicitly takes into account network effects, the historical spatial distribution of people and road transport supply. The used accessibility measures are split into a base accessibility level, and an additional level obtained by fast main roads. Historical changes over the last 50 years in most municipalities of 10 countries in mainland Western Europe are investigated. Those provide a considerable amount of observations with differently timed moments of economic integration but relatively similar economic regimes, compared to other parts of the continent, such as the former Warsaw Pact countries that comprised Eastern Europe during most of the studied period. Section 2 discusses the method to decompose accessibility, the selected study area and the data applied in the analyses. Section 3 discusses accessibility trends and the potential role of EU policies in foreign accessibility. Section 4 discusses the effect of cross-border spillovers on the historical distribution of growth. We end with recommendations regarding the role of transport investment in a process of international economic integration in Section 5.

## 2 Accessibility decomposition and applied data

At the basis of this study is the decomposition of a potential accessibility measure into the level of accessibility offered by a slow base network and the additional interaction opportunities offered by Europe's highways. Highways are meant here in their colloquial sense, comprising main thoroughfares and motorways, and are observed in a historical dataset that has recently become available (Stelder et al., 2013). The base and highway-induced components of accessibility are subsequently split into domestic (DOM) and foreign (FOR) components, leaving in total four components, of which foreign base accessibility and foreign highway-induced accessibility indicate cross-border accessibility spillovers. The separated accessibility components are used to find to what degree cross-border transport supply lags behind compared to domestic transport supply, and if so, whether EU membership coincides with reductions in the gap between domestic and transport supply. Such reductions might be caused by TEN-T policies that are in part set up in the 1990s to improve cross-border accessibility between EU member states (van Exel et al., 2002). The introduced variables are subsequently used to evaluate the impact that cross-border market access may have had on population growth.

### 2.1 Accessibility computation

All accessibility components used in this paper share their origin in a potential accessibility measure. It is computed as:

(1) $$A_{i,t}=\sum_{i\neq j}^{n}P_{j,t}{\left(M_{\mathrm{ij},t}\right)}^{\gamma },$$

so that accessibility *A* for origin municipality *i* is computed based on, on the one hand, the population counts *P* in destination municipalities *j* in decade *t*, and on the other hand distance-decayed travel-times in minutes in *M*. The travel times are produced by a shortest path finding algorithm applied on a transport network with assumed travel speeds in decade *t*. It is important to note that in the accessibility measure used here, intra-zonal accessibility levels have been discarded so that this study deals only with accessibility to external destinations. This somewhat unconventional choice is made because the inclusion of best-guessed internal interaction opportunities is not necessary in this study of transport network effects on population growth. In fact, including internal interaction opportunities caused substantial collinearity issues with urban densities in the analysis presented in Section 4. That internal interaction opportunities need not be estimated is fortunate, as validated methods to estimate real internal travel times were unavailable at the time this paper was prepared (Stepniak and Jacobs-Crisioni, 2017) and any guess of internal interactions was thus unsure.

A crucial aspect of this approach is the selection of distance decay functions that characterise the average dislike of distance in travelling. Because an empirically obtained European distance decay parameter is missing, the study focuses on accessibility measures with a relatively steep distance-decay, so that *γ* = − 2, which implies that the accessibility measures emphasize proximate population masses. This distance decay function is chosen because, similar to other studies (Jacobs-Crisioni et al., 2014), it yielded the best explained variance in the later population change models. Results with *γ* = − 1.5 are presented in the appendices; *γ* = − 1 has been tested as well, but the resulting accessibility components proved too collinear for reliable estimation of the population growth model.

### 2.2 Selection of origins and destinations

With regard to the analyzed origin zones *i*, the study area covers most of mainland Western Europe, including all original EU member states (Belgium, France, Germany, Italy, Luxembourg and the Netherlands), the 1986 EU additions (Portugal and Spain) and one 1995 EU addition (Austria). The study area and years of accession are mapped in Fig. 2. Non-EU member Switzerland is included as well. Switzerland is included to control for the effects of EU membership; but we must acknowledge that it is presumably relatively open to surrounding EU member states, as Switzerland is a member of the European Economic Area, and has many economic and political ties with the EU.

Cross-border market integration is chiefly relevant for border municipalities. However, an a-priori selection of the municipalities that might be affected by border effects could substantially bias the results. Explicitly taking foreign market access into account in the analytical framework undoes the need to do such an a-priori selection, so we can use the entire set of municipalities in a country here. To test whether the conclusions hold if only border municipalities are analyzed, the analyses have been repeated on all municipalities in the study area that are maximally 30 km away from national borders.

Germany is a special case in Europe's recent history, as between 1949 and 1990 the current country was split into separate western and eastern states with contrasting economic systems and political affiliations (Redding and Sturm, 2008). To maintain political and economic homogeneity, only the municipalities in former West Germany are selected as origins in this study. Before 1990, destinations in former East Germany are considered as foreign for municipalities in West Germany; they are considered as domestic destinations thereafter. The German unification had no clear impact on the analyses presented in this article. Because of the stark economic separation that existed between Western and Eastern Europe until roughly 1990, all destinations in former Warsaw Pact countries are ignored in the results presented in the main article. Analyses with accessibility measures that included destinations in those countries showed that this choice does not affect this paper's conclusions.

Because of waiting times and additional financial costs, ferries presumably make interaction more costly than the travel times in *M*_*ij* , *t*_ indicate. This makes an accurate measure of interaction opportunity difficult in places that are highly dependent on ferries. Therefore all of the selected countries' islands in the Atlantic and Mediterranean are excluded except for the sizeable islands of Corsica, Sardinia and Sicily. The latter islands are expected to be at least partially autonomous, so that the dependence on hard-to-model ferry services is less crucial for total market access. Lastly, because of their particular economic and political character, the microstates Andorra, Liechtenstein, Monaco, San Marino and Vatican City are not taken into account as origins. As destination zones *j* all municipalities within 5 h travel time from an origin are included in the travel-time matrix that governs the computation of accessibility values. In order to test the robustness of the presented results, the analyses have been repeated with a 2.5 h cut-off.

Lastly, it is important to note that the observations are weighted by the area of their areal units. This is done to reduce the impact of differences in geographical size (Jacobs-Crisioni et al., 2014), and is particularly necessary in this paper because areal unit sizes differ substantially per country; not weighting would cause a substantial overrepresentation of observations in countries with many small municipalities such as Italy and France. Additional tests have shown that this causes unexpected outcomes from the population growth model in particular in the group of 1957 accession countries, which have the largest variance in municipal size.

### 2.3 Base and highway-induced accessibility components

As shown by Koopmans et al. (2012), accessibility in *A* combines the element of centrality, or the relative position of a municipality with regard to the spatial distribution of activities; and the element of transport network service, or potential alterations from centrality due to travel time reductions offered by the existing transport network. We define a similar measure of centrality as total base accessibility *B*:

(2) $$B_{i,t}=\sum_{i\neq j}^{n}P_{j}{\left(M_{\mathrm{ij},t}^{\mathrm{base}}\right)}^{\gamma },$$

so that total base accessibility depends on the existing population distribution in *P* and the travel times in matrix *M*_*ij* , *t*_^*base*^. Koopmans et al. (2012) define the travel times in this matrix as the travel times before a new transport mode starts to expand. In this study, *M*_*ij* , *t*_^*base*^contains travel times from the modelled base networks, so that *B* contains accessibility levels if all municipalities were only connected by means of a well-connected but relatively slow road network. The speeds on the base road networks in a decade are linked with the speeds on the observed network from that decade, so that general speed improvements on Europe's road networks due to better roads or faster cars are taken into account in this measure.

With the computed base accessibility component, we can compute the value that faster transport network services have added to total accessibility levels. In this study we define this as a measure of highway-induced accessibility levels in *T*:

(3) $$T_{i,t}=A_{i,t}-B_{i,t}.$$

Thus, the total highway-induced accessibility component *T* indicates the augmenting effect that higher speeds on the observed highways have on overall accessibility levels. It is important to emphasize that *B* and *A* depend on the same set of destinations, so that changes in population levels at the destination are controlled for in *B*. This is relevant for the subsequent analyses.

### 2.4 Domestic and foreign components

To be able to measure the impact of domestic and foreign accessibility components, the accessibility values are further separated by discerning the destinations in the accessibility computations using a dummy variable. Thus, let *F*_*ij* , *t*_ = 1 only if *i* and *j* are in two separate countries in a specific decade, else *F*_*ij* , *t*_ = 0. Subsequently separate domestic and foreign accessibility levels can be computed:

(4.1) $$A_{i,t}^{\mathrm{DOM}}=\sum_{i\neq j}^{n}\left(1-F_{\mathrm{ij},t}\right)P_{j}{\left(M_{\mathrm{ij},t}\right)}^{\gamma },$$

(4.2) $$A_{i,t}^{\mathrm{FOR}}=\sum_{i\neq j}^{n}F_{\mathrm{ij},t}P_{j}{\left(M_{\mathrm{ij},t}\right)}^{\gamma },$$

It must be clear to the reader that a further decomposition of these accessibility values into their *B* and *T* components can be achieved through the (2), (3), so that we will not describe the generation of domestic base accessibility *B*_*i* , *t*_^*DOM*^, domestic highway-induced accessibility  *T*_*i* , *t*_^*DOM*^, foreign base accessibility *B*_*i* , *t*_^*FOR*^ and foreign highway-induced accessibility *T*_*i* , *t*_^*FOR*^ explicitly here. For the sake of completeness the definition of these variables is given in Appendix I.

### 2.5 Data

The local population dataset used here is the combined product of many national statistical offices and describes population counts in Europe between 1961 and 2011 for each decade (Gløersen and Lüer, 2013) as they would be within the 2011 municipal borders. These population counts are derived from censuses for all municipalities. Where municipal borders have changed in the covered period, the census data has been subject to aggregation and/or spatial interpolation to ensure that the observed population counts are consistent with the 2011 municipal borders. The necessary spatial interpolation might have caused slight misallocations between neighbouring municipalities. We do not expect that these misallocations will affect the reported results substantially, the more so because the paper focuses on accessibility levels and population densities, which are relatively insensitive to issues of spatial delineation (see Arbia, 1989). The used dataset all in all provides a highly detailed account of population levels and population changes in Europe in the last 50 years. Summary population changes in the study area are given in Table 1.

**Table 1:** Population and population changes by groups of countries with different accession years.

	1961	1971	1981	1991	2001	2011
Population (millions)						
EU members since 1957	170.9	186.2	194.3	200.2	207.6	215.9
EU members since 1986	37.4	40.3	44.7	46.3	47.9	53.0
EU member since 1995	7.1	7.5	7.6	7.8	8.1	8.5
Switzerland	5.4	6.3	6.4	6.9	7.3	7.9

Population growth (1961 = 100)						
EU members since 1957	100	109.0	113.7	117.2	121.5	126.3
EU members since 1986	100	107.9	119.6	123.8	128.1	141.8
EU member since 1995	100	105.8	106.9	110.3	113.7	119.1
Switzerland	100	115.5	117.3	126.6	134.3	146.2

The main transport network for each decade between 1960 and 2010 is taken from a recent dataset that describes the development of main roads in Europe (Stelder, 2016). These data yield a relatively dense network of main national roads, motorways and ferry services with associated travel speeds (Table 2). Changes in the service level of the observed roads may thus come from links that were newly built in that decade, or because of recorded changes in the travel speed on the observed links.

**Table 2:** Travel speed distribution on highways (in km/h) as mapped by Stelder et al. (2013).

Year	5th perc	Average	Max
1961	48.2	56.9	83
1971	51.0	67.0	96
1981	53.2	71.5	98
1991	55.4	73.6	103
2001	56.8	75.0	108
2011	59.3	82.8	113

**Fig. 1:**
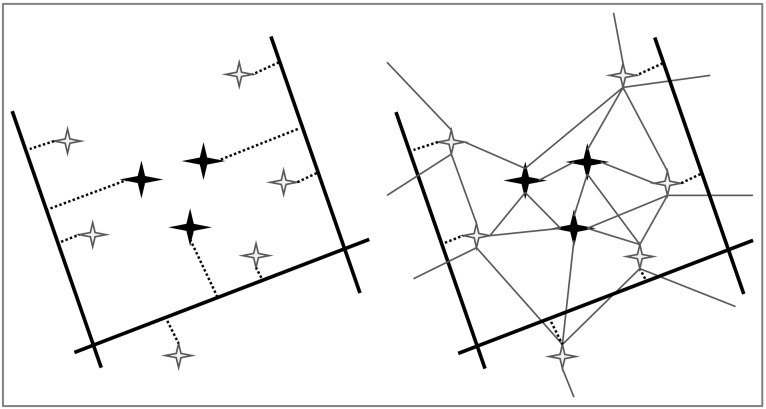
Methods to ensure that zones are connected to a transport network. On the left the common method, with all zones (star shapes) connected to the closest highway (thick black lines) by means of connector links (dashed lines); on the right the approach applied in this article in which all zones are connected through a modelled underlying network (thin lines) and only zones that have a highway in their territory are directly connected to the highway network by means of connector links.

**Fig. 2:**
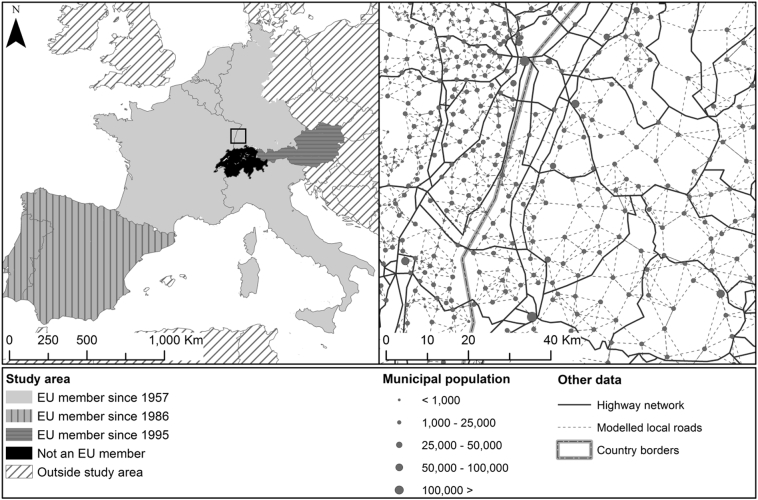
Study area and years of EU accession of the studied countries (left); population distribution and highway network as mapped by Stelder et al. (2013) in 2011 (right). The rectangle in the middle of the left map indicates the geographical coverage of the map on the right.

Despite the detail in the highways dataset, many municipalities in the population database are not close to any highway. The issue of such distant zones is commonly solved by modelling long connector links between observed roads and distant zones. However, this may lead to biases in the computed accessibility measures, for example when a zone may be expected to be served almost equally by two parallel roads but is only connected to one of both. Such a situation may cause an underestimation of accessibility to particular destinations and directions. Collecting a comprehensive European road network spanning multiple decades is impractical. To tackle this problem, the observed main road network is therefore augmented with a modelled network that serves to approximate the unobserved underlying local road network. This network is modelled by linking every municipality to its five nearest neighbours within the same NUTS3 region, and the one closest municipality from neighbouring NUTS3 regions if that exterior municipality is closer than the farthest of the selected five interior neighbours. The result is a relatively sparse network that, at least in continental Europe, provides a direct connection between all observed municipalities. Only the municipalities that have a highway in their territory are subsequently connected to the highways network. In the modelled underlying network, all connections are by straight lines. The common method to model zone connections and the approach applied in this article are illustrated in Fig. 1. Fig. 2 shows the study area and the detail of the used municipal data and road networks in the region around the German-French border.

The 5th percentile observed travel speed on the main road network in a given decade is imposed on the entire underlying road network, so the transport speeds on the modelled local road network can be considered to be relatively slow. When using the assumed speeds and link lengths to compute the time needed to traverse a link, link lengths are multiplied by 1.2 to take into account a general small degree of circuity that is not taken into account in the method for modelling roads. We must acknowledge that this method is only a very rough approximation of the real underlying travel times; effective travel speeds may vary much more. Such variation may exist because of, for example, local network improvements or limitations imposed by physical geography. We are nevertheless confident that this modelled network yields less biased travel time estimates than the alternative method of connecting all municipalities directly to main roads.

### 2.6 Computed municipal accessibility values

**Fig. 3:**
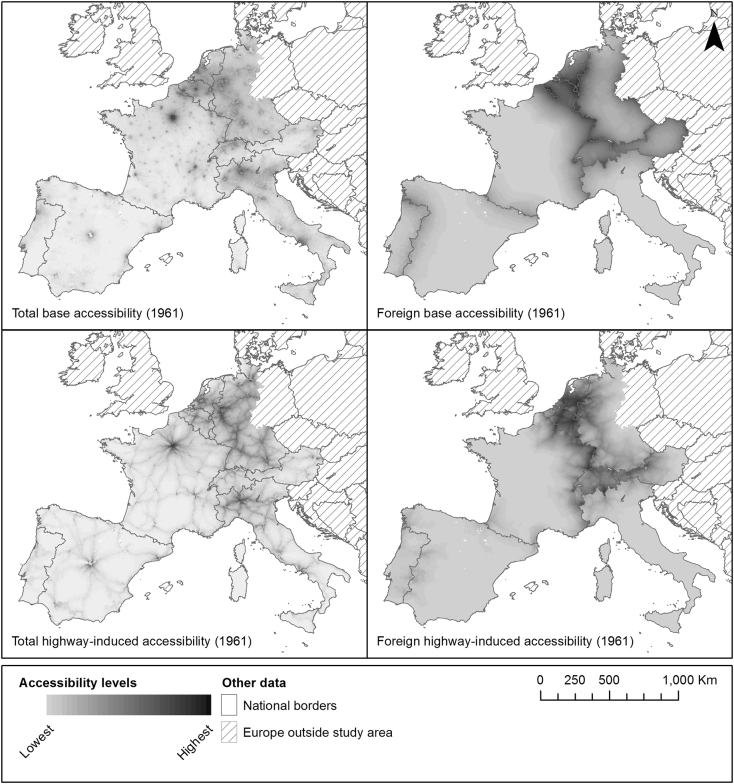
Total and foreign base and highway-induced accessibility values in 2011 in the study area. The foreign base and highway-induced accessibility values are expressed as a percentage of, respectively, total base and total highway-induced accessibility levels.

The accessibility computations have been executed using the open-source GeoDMS software (ObjectVision, 2014). By way of example, the computed, decomposed accessibility values for one decade (1961) are shown in Fig. 3. The total (domestic and foreign) base accessibility patterns demonstrate the predominance of Europe's metropolitan regions in accessibility levels. When comparing total base accessibility with foreign base accessibility, it is clear that in Europe, foreign population distributions have a very limited impact on total base accessibility levels. Only in the northern and north-eastern border areas of France and in the border areas between Belgium, Germany and The Netherlands, total base accessibility levels are visibly affected by base foreign accessibility.

As can be expected, the highway-induced accessibility patterns emphasize the main transport corridors in Europe. But this extensive highway network also brings fast overland interaction opportunities to relatively peripheral regions such as the centre of France. Again, when comparing total highway-induced accessibility with its foreign component, it is apparent that cross-border interaction opportunities had a limited impact on total accessibility levels in Europe's larger countries. In smaller countries, foreign interaction opportunities were much more substantial, confirming the previously noted often asymmetric nature of international accessibility spillovers (Hansen, 1977). The faster speeds on the highway system allow for a much deeper penetration of foreign interaction opportunities than the base accessibility levels. Nevertheless, compared with foreign base accessibility levels, the patterns remain similar, with the same countries enjoying the highest foreign highway-induced accessibility.

## 3 Is cross-border transport supply lagging behind?

This study tackles two questions that deal with cross-border transport infrastructure supply. First of all, has growth in cross-border transport supply lagged behind domestic transport supply? And secondly, has ongoing international economic integration had an impact on cross-border transport supply? To answer these questions, the results of the accessibility computations have first been aggregated into average values for groups with different years of accession to the EU and Switzerland.

The results of this exercise are shown in Table 3. A number of conclusions can be obtained from these results. A first finding is that the groups had substantially differing starting positions. These starting positions reflect a number of aspects, including the relative position of the studied countries with regard to Europe's population and the level of transport service offered within those groups. In all groups, accessibility values have tripled. Initial values do not seem to have had an impact on the growth of accessibility levels though; the two largest growing groups are the 1986 members, which had the lowest initial values, and Switzerland, the country outside of the EU that had the highest initial value. From changes in the highway-induced accessibility component it becomes clear that a substantial part of the historical changes in accessibility have been caused by improvements in Western Europe's main transport network. In all groups, growth in highway-induced accessibility far outpaces total accessibility increases and population growth (see Table 1). Thus, in the last 50 years, interaction opportunities in Western Europe have become increasingly dependent on Europe's highways.

**Table 3:** Historical averaged base and highway-induced accessibility levels.

	Average values		% of potential accessibility		Index (1961 = 100)		
	1961	2011	1961	2011	1971	1991	2011
*Potential accessibility*							
In EU since 1957	193.6	575.6			138.3	206.7	297.3
In EU since 1986	64.6	229.8			124.4	193.8	355.9
In EU since 1995	123.9	369.8			129.8	198.9	298.6
Not an EU member	205.9	674.2			145.0	221.1	327.4

*Total base accessibility*							
In EU since 1957	160.1	337.1	82.7	58.6	120.3	158.2	210.6
In EU since 1986	56.7	127.5	87.8	55.5	118.3	163.5	224.9
In EU since 1995	106.3	223.4	85.8	60.4	119.4	156.4	210.2
Not an EU member	175.1	418.6	85.0	62.1	127.1	169.6	239.1

*Total highway-induced accessibility*							
In EU since 1957	33.5	238.4	17.3	41.4	224.1	438.4	711.9
In EU since 1986	7.9	102.3	12.2	44.5	168.5	411.7	1299.8
In EU since 1995	17.6	146.4	14.2	39.6	192.6	456.2	833.0
Not an EU member	30.9	255.6	15.0	37.9	246.1	513.3	827.8

*Foreign base accessibility*							
In EU since 1957	14.2	39.8	7.4	6.9	129.4	185.0	279.7
In EU since 1986	2.9	7.0	4.5	3.1	111.3	159.5	242.1
In EU since 1995	16.3	82.7	13.2	22.4	129.3	184.9	505.9
Not an EU member	48.6	126.9	23.6	18.8	131.2	184.3	261.1

*Foreign highway-induced accessibility*							
In EU since 1957	4.2	52.1	2.2	9.1	284.7	718.9	1226.6
In EU since 1986	0.4	8.2	0.7	3.6	154.6	457.6	1837.4
In EU since 1995	5.9	83.3	4.8	22.5	209.8	472.6	1404.6
Not an EU member	15.7	136.8	7.6	20.3	252.0	535.7	872.6

With regard to foreign highway-induced accessibility, again, the initial values vary substantially, and here, initial values do seem to have affected levels of growth: the countries with the lowest initial values display the largest growth. Clearly, relatively small landlocked countries such as Austria (member since 1995) and Switzerland are much more dependent on foreign interaction opportunities than sizeable, relatively isolated countries such as the 1986 members Portugal and Spain. In fact, in Austria and Switzerland, foreign highway-induced accessibility contributes more to total accessibility levels than its domestic counterpart: e.g. in Switzerland, 20.3% of the total potential accessibility value is contributed by foreign highway-induced accessibility, so that the remainder domestic highway contribution is 17.6%. When comparing growth in the various accessibility components, in all groups, foreign highway-induced accessibility has grown more than total highway-induced accessibility. Thus, cross-border transport supply is clearly catching up with domestic transport supply.

Did EU membership coincide with larger growth in cross-border transport supply? Comparing growth rates and relative contributions of the foreign transport component seems to corroborate that. Table 3 shows that all EU members have had higher growth rates than Switzerland. In 1961, Switzerland had the highest contribution of foreign highway-induced accessibility in total accessibility. In 2011 that top position has been taken over by 1995 member Austria. Furthermore, until 1991, both Austria and the 1986 accession countries have lagged behind Switzerland and the 1957 accession countries in terms of growth of cross-border transport supply. Thus, between 1991 and 2011, growth in highway-induced foreign accessibility in Austria, Spain and Portugal has increased in pace dramatically, coinciding with the EU accession of these countries and with the start of the EU's TEN-T policies in 1991. This coincidence is conspicuous, but may be caused by the fact that recently, major infrastructural investments in Switzerland were dedicated to rail infrastructure, such as the partially EU-funded AlpTransit project. An in-depth study of the effects of infrastructure projects supported by EU policies is needed to proof causation.

We must emphasize that the presented results have also been computed without area weighting, with shorter maximum travel times, only for municipalities 30 km away from a border and for accessibility values that include former Warsaw Pact countries as destinations; all alternatives support the same qualitative findings.^2^ An important finding is that in the 1957 and 1995 accession countries, growth in average foreign highway-induced accessibility is reduced by 25% and 33% respectively, when municipalities in Warsaw Pact countries are included as destinations. This clearly signals the lack of road network improvements between the Western and Eastern Bloc countries in the observed period. Another noteworthy finding is that, for municipalities close to national borders, growth in highway-induced accessibility to foreign destinations is less pronounced. Here, no doubt because of the relatively good starting position of municipalities at the border, inland municipalities profited more from improved highway access to foreign destinations.

The fact that cross-border connectivity has improved drastically, begs the question if it ever was at a disadvantage? Given the impact that geography may have on accessibility distributions, comparing averages is not useful here. Because transport supply generally follows transport demand (Jacobs-Crisioni and Koopmans, 2016, Xie and Levinson, 2011), a more suitable comparison of the performance of domestic and foreign transport supply should take into account to what degree these satisfy demand for travel. If cross-border highway-induced accessibility was at a disadvantage at the start, one may expect that cross-border demand had a smaller impact on foreign highway-induced accessibility than is the case with domestic demand and domestic connectivity. To test this, we used domestic and foreign base accessibility levels as proxies of potential demand; the usefulness of those proxies is supported by empirical evidence (Kolars and Malin, 1970, Warntz, 1966). A cross-sectional regression method has been applied to find to what degree domestic and cross-border connectivity are linked to potential demand. The expectation is that, if more people are geographically closer, it is more likely that they are served better by the main road network. The following equations were fitted:

(5.1) $$T_{i\in k,t=1961}^{\mathrm{DOM}}=\beta 0_{k}+\beta 1_{k}B_{i\in k,t=1961}^{\mathrm{DOM}}+\beta 2_{k}B_{i\in k,t=1961}^{\mathrm{FOR}}+\varepsilon_{i};$$

(5.2) $$T_{i\in k,t=1961}^{\mathrm{FOR}}=\beta 0_{k}+\beta 1_{k}B_{i\in k,t=1961}^{\mathrm{DOM}}+\beta 2_{k}B_{i\in k,t=1961}^{\mathrm{FOR}}+\varepsilon_{i};$$

so for all municipalities in the study area the effects of domestic and foreign base accessibility are estimated on both domestic and foreign highway-induced accessibility in 1961. These equations are fitted separately per group of municipalities within the countries *k* that share a similar EU accession date. As noted before, self-potential of the studied municipalities is excluded here, so that in both models only external accessibility values are studied. Thus, the results are not inherently biased because of the logical omission of intra-zonal interaction opportunities in foreign accessibility. The results are given in Table 4.

**Table 4:** Results of regressions that explore linkages between highway-induced accessibility levels and domestic (dom.) and foreign (for.) base accessibility levels in 1961.

	Effect on domestic highway-induced accessibility		Effect on foreign highway-induced accessibility	
	Coef.	(t-Score)	Coef.	(t-Score)
*EU members since 1957*				
Base dom. accessibility	0.20⁎⁎	(218.93)	0.01⁎⁎	(40.51)
Base for. accessibility	− 0.06⁎⁎	(− 10.83)	0.23⁎⁎	(276.96)
Constant	0.01⁎⁎	(3.35)	0.01⁎⁎	(25.48)
N	54,296		54,296	
R^2^	0.47		0.60	

*EU members since 1986*				
Base dom. accessibility	0.11⁎⁎	(45.85)	0.00⁎⁎	(8.48)
Base for. accessibility	− 0.29⁎⁎	(− 9.96)	0.10⁎⁎	(72.87)
Constant	0.03⁎⁎	(11.74)	0.00⁎⁎	(9.27)
N	8237		8237	
R^2^	0.22		0.39	

*EU member since 1995*				
Base dom. accessibility	0.13⁎⁎	(28.01)	0.00	(0.5)
Base for. accessibility	0.00	(0.34)	0.21⁎⁎	(41.62)
Constant	− 0.01	(− 1.50)	0.02⁎⁎	(9.99)
N	2361		2361	
R^2^	0.25		0.42	

*Not an EU member*				
Base dom. accessibility	0.10⁎⁎	(31.72)	0.00	(0.98)
Base for. accessibility	0.01	(0.33)	0.27⁎⁎	(61.0)
Constant	0.02⁎	(2.07)	0.02⁎⁎	(9.55)
N	2539		2539	
R^2^	0.29		0.60	

Coefficients are noted as coef.

⁎ p < 0.05.

⁎⁎ p < 0.01.

The results show that, as expected, potential demand for travel as proxied by base accessibility has a positive effect on highway-induced accessibility. Thus, domestic highway-induced accessibility is higher where domestic base accessibility is higher, and foreign highway-induced accessibility is higher where foreign base accessibility is higher. Surprisingly, according to these results, in all groups except for the 1986 accession countries, domestic highway-induced accessibility levels are lower per unit of domestic base accessibility than is the case with foreign highway-induced accessibility and foreign base accessibility levels. One explanation is that the perspective of potentially lucrative international fares has caused that foreign destinations are relatively well served; a similar result has been found in the context of the Dutch railway network (Jacobs-Crisioni and Koopmans, 2016). All in all, these results do not support the expectation that cross-border connectivity lagged behind domestic connectivity. If nevertheless cross-border transport supply was poor in border areas, the reason must lie in poor cross-border travel demand, for example due to structurally lower population levels in border regions.

## 4 Are borders a barrier to population growth?

Now it is clear that cross-border connectivity has increased drastically in West Europe in the last 50 years, the remaining question tackled here is whether cross-border market access has in fact affected the spatial distribution of population growth in Europe? And is cross-border market access more relevant for population growth in EU member states? To explore this question the following equation is fitted:

(6) $$∆PD_{i\in k,t}=\beta_{0k}+\beta_{1,i\in k}+\beta_{2k,t}+\beta_{3k,t}B_{i\in k,t-1}^{\mathrm{DOM}}+\beta_{4k,t}B_{i\in k,t-1}^{\mathrm{FOR}}+\beta_{5k,t}T_{i\in k,t-1}^{\mathrm{DOM}}+\beta_{6k,t}T_{i\in k,t-1}^{\mathrm{FOR}}+\alpha_{d,k,t}PD_{i\in k,t-1}X_{d}+\varepsilon_{i,t},$$

in which ∆* PD*_*i* ∈ *k* , *t*_ contains absolute changes in population densities defined as $PD_{i,t}-PD_{i,t-1}=\frac{P_{i,t}}{{\mathrm{Area}}_{i}}-\frac{P_{i,t-1}}{{\mathrm{Area}}_{i}}$ per group of countries *k* that accessed the EU in the same year. Changes in population densities are used as the dependent variable here, as it is well-known that density-based variables are less sensitive to biases resulting from the Modifiable Areal Unit Problem (Arbia, 1989, Briant et al., 2010). *β*_1 , *i* ∈ *k*_ contains a fixed effect for each municipality; *β*_2k , t_ contains an estimator for each year; *B*_*i* ∈ *k* , *t* − 1_^*DOM*^ and *B*_*i* ∈ *k* , *t* − 1_^*FOR*^ contain, respectively, domestic and foreign base accessibility; *T*_*i* ∈ *k* , *t* − 1_^*DOM*^ and *T*_*i* ∈ *k* , *t* − 1_^*FOR*^ contain, respectively, domestic and foreign highway-induced accessibility in the prior decade; and lastly, *PD*_*i* ∈ *k* , *t* − 1_*X*_*d*_ contains prior population densities, with the effect of prior population densities estimated separately for different value ranges *d*. The separation into different value ranges is done in order to allow for a nonlinear relationship that may be the result of interactions between agglomeration benefits and congestion effects. For all variables, values at the start of a decade are used to explain changes in population densities in that decade. This is done to disentangle the effect of accessibility on population changes from the reciprocal effect that population changes have on network improvements and population distribution, so that endogeneity issues that are common in studies of the effect of transport investments (Percoco, 2015) can be partially avoided. We must acknowledge that the case that population growth has preceded expected accessibility levels cannot be excluded using this method. Recent evidence shows that substantial accessibility improvements by planned infrastructure projects have caused anticipatory effects in housing prices only in the very last years before the new infrastructure is opened (Hoogendoorn et al., 2016). It may be assumed that population changes are even later. Because this study captures differences per decade, we therefore assume that the effect of anticipation will be limited in the presented results.

A fixed-effect method has been applied here in which a time-invariant coefficient is estimated for each municipality separately. The influence of static geography on municipal growth is captured implicitly in those fixed effects. Hausman tests justify the adoption of a fixed-effect approach. Time-specific effects are deliberately included to account for likely heterogeneity over time in the observed data, such as may be caused by large scale events such as the oil crisis in the 1970s and the reunification of West and East Germany in the 1990s. The choice for time-specific effects is supported by Wald tests. The effects of the constant values, centrality and connectivity aspects of the estimation are given in Table 5; the prior population density effects and the estimated fixed effects are shown in Appendix II. Lastly, a Harris-Tzavalis unit root test has been executed to verify whether the assumption of temporal stationarity holds with this modelling exercise; the results indicated likely temporal stationarity of the studied processes.

**Table 5:** Results of base and highway-induced accessibility components on population density changes.

	In EU since 1957	In EU since 1986	In EU since 1995	Not an EU member
*General constant*	63.61⁎⁎ (51.95)	14.33⁎⁎ (7.29)	6.81⁎⁎ (3.36)	− 16.80⁎⁎ (− 2.64)
1971	0.00	0.00	0.00	0.00
1981	− 0.27 (− 0.47)	− 3.41⁎⁎ (− 3.37)	− 1.59⁎⁎ (− 2.90)	0.40 (0.20)
1991	− 3.82⁎⁎ (− 6.55)	4.23⁎⁎ (4.09)	− 3.15⁎⁎ (− 5.59)	6.55⁎⁎ (3.07)
2001	− 4.76⁎⁎ (− 8.02)	− 0.91 (− 0.87)	− 4.21⁎⁎ (− 7.31)	4.82 (1.95)
2011	− 3.97⁎⁎ (− 6.54)	− 3.17⁎⁎ (− 2.90)	− 4.90⁎⁎ (− 7.19)	11.27⁎⁎ (4.17)
*Prior domestic base accessibility*				
1971	4.89⁎⁎ (8.13)	28.55⁎⁎ (11.77)	8.96⁎⁎ (5.56)	59.03⁎⁎ (22.55)
1981	5.54⁎⁎ (10.97)	23.43⁎⁎ (14.70)	11.61⁎⁎ (8.45)	48.06⁎⁎ (23.53)
1991	8.56⁎⁎ (18.83)	11.34⁎⁎ (9.46)	10.39⁎⁎ (8.32)	42.97⁎⁎ (22.88)
2001	8.22⁎⁎ (20.18)	18.47⁎⁎ (17.18)	9.15⁎⁎ (8.29)	39.89⁎⁎ (24.41)
2011	8.55⁎⁎ (23.66)	16.83⁎⁎ (17.07)	7.75⁎⁎ (7.85)	35.47⁎⁎ (25.16)
*Prior foreign base accessibility*				
1971	− 37.21⁎⁎ (− 8.04)	− 29.78 (− 0.75)	1.79 (0.69)	31.26⁎ (2.40)
1981	− 33.29⁎⁎ (− 8.60)	7.02 (0.19)	− 0.79 (− 0.35)	27.06⁎⁎ (2.78)
1991	− 24.53⁎⁎ (− 6.95)	− 28.50 (− 0.87)	1.29 (0.62)	22.45⁎⁎ (2.76)
2001	− 24.09⁎⁎ (− 7.50)	− 1.37 (− 0.04)	1.91 (1.04)	30.48⁎⁎ (4.43)
2011	− 22.62⁎⁎ (− 8.21)	4.85 (0.17)	1.83* (2.02)	27.59⁎⁎ (4.64)
*Prior domestic highway-induced accessibility*				
1971	1.82⁎ (2.22)	− 48.08⁎⁎ (− 10.88)	10.38⁎⁎ (6.54)	79.80⁎⁎ (21.58)
1981	0.68 (1.38)	11.30⁎⁎ (4.64)	2.11 (1.61)	28.60⁎⁎ (12.42)
1991	5.85⁎⁎ (14.55)	− 2.87 (− 1.79)	6.15⁎⁎ (6.20)	18.88⁎⁎ (9.55)
2001	4.38⁎⁎ (12.68)	1.20 (0.89)	8.44⁎⁎ (10.78)	16.30⁎⁎ (10.20)
2011	0.55 (1.84)	5.05⁎⁎ (5.58)	8.92⁎⁎ (13.20)	17.16⁎⁎ (12.33)
*Prior foreign highway-induced accessibility*				
1971	− 22.70⁎⁎ (− 3.98)	− 95.66 (− 1.14)	− 11.12 (− 1.55)	16.18 (1.08)
1981	− 3.69 (− 1.40)	− 173.27⁎ (− 2.53)	1.40 (0.34)	1.21 (0.18)
1991	− 7.83⁎⁎ (− 4.57)	− 65.39 (− 1.87)	0.98 (0.32)	− 11.46 (− 1.86)
2001	− 1.11 (− 0.75)	− 79.90⁎⁎ (− 2.98)	0.46 (0.20)	− 22.07⁎⁎ (− 4.52)
2011	− 0.76 (− 0.61)	− 62.76⁎⁎ (− 3.78)	− 1.02 (− 0.61)	− 29.88⁎⁎ (− 6.93)
N	271,475	41,185	11,805	12,695
R^2^	0.40	0.67	0.82	0.70

⁎ p < 0.05.

⁎⁎ p < 0.01.

This analysis yields a number of interesting findings. A first result is that in all groups of countries, domestic base and domestic highway-induced accessibility levels have had a significant and positive effect on changes in population density in the study area, although, when compared with the results of prior population densities, the signal of accessibility effects is less clear. Nevertheless we find that, in the last 50 years, municipalities with better base and highway-induced accessibility to domestic destinations have consistently grown more. This supports the often repeated result that market access, enabled by investments in infrastructure, have had a positive effect on population growth (Hansen, 1959, Koopmans et al., 2012).

In all country groups, only accessibility to domestic destinations has had a positive effect on municipal population growth. This confirms clearly that, in the study area, borders have and are limiting population growth through a persistent reduction of relevant market access (Brakman et al., 2012, Redding and Sturm, 2008). Highway-induced accessibility to foreign destinations has not had a significant positive impact in any of the studied groups, despite of the large improvements reported in Section 3. Only in Austria and Switzerland an insignificant positive can be noted here for specific years. If increasing market access through better cross-border road connections has not clearly improved the position of border regions in the studied country groups, this casts doubt on the efficacy of cross-border transport network investments as a means to further integrate local markets across borders. Furthermore, given the substantial contribution of foreign highway-induced accessibility found in this paper and reported by Stępniak and Rosik (2013), these findings imply that substantial potential for growth is underutilized in particular in Europe's border regions.

To verify the robustness of these findings, this analysis has been repeated with different distance decay functions; without area weighting; with shorter maximum travel times; only for municipalities 30 km away from a border; and for accessibility values that include former Warsaw Pact countries as destinations. All results support the same conclusions.^3^ Thus, foreign highway-induced accessibility has in no case caused structural increases in population density. Other noteworthy findings from these alternative specifications is that with *γ* = − 1.5, the effects of foreign base accessibility are much reduced; that foreign base accessibility has had a positive, although usually not statistically significant effect on population changes specifically in border areas; and that for West-German municipalities, the effect of base foreign accessibility changes from negative to positive when including destinations in former Warsaw Pact countries. The latter finding may indicate that municipalities in West Germany still had ties or shared factors of growth with neighbouring East-German municipalities.

One may expect that accession to the EU has affected local population growth. This has been shown before for border cities and border regions, even though positive effects of EU enlargement have been relatively small compared to the negative effects of being close to a border (see Brakman et al., 2012). The present results do not demonstrate a clear coincidence between foreign accessibility effects and EU membership; on the contrary, only Switzerland yields a positive effect of foreign base accessibility. A potential explanation for the differences with previously found EU effects may be in the inclusion of all municipalities in this study. Brakman et al. (2012) find that only large cities or regions in border regions benefitted clearly from EU enlargements, while smaller cities and regions were not significantly affected. This could to some degree be repeated with the presented data by selecting only large municipalities.^4^ An indepth study will be needed to more accurately replicate Brakmans's findings with the presented data; we consider such an exercise outside of the scope of this paper.

Lastly, only a small portion of population density changes can be explained through accessibility levels. Prior population densities and the model's fixed effects explain a great deal of the variance in population density changes. The found fixed effects highlight existing urban and metropolitan areas in the study area and in fact have a very high (0.89–0.94) positive correlation with population densities, regardless of the observed year. Our interpretation of this result is that the geographic distribution of many of the factors that define municipal attractiveness, such as natural endowments, the presence of services and the availability of employment, has been relatively static in the observed time period. The attractiveness observed in the fixed effects is offset by prior population densities, which have had a substantial and lasting limiting impact on municipal growth. This corroborates the findings presented by Koopmans et al. (2012). The effect of prior population densities on density change is very non-linear, with generally least negative effects around 250 persons per km^2^. Lower population densities are in general less attractive, possibly due to a lack of typical agglomeration benefits such as availability of jobs and services. Higher population densities are also less attractive, possibly due to negative externalities such as housing market congestion and pollution.

## 5 Conclusions and discussion

This paper presents an analysis of the development of accessibility and population growth in Western Europe between 1961 and 2011. It finds that, largely due to improvements to Europe's highway network, interaction opportunities in the studied area have improved more than its population has grown. Interaction opportunities across borders have increased even more than total accessibility levels, in particular in EU member states. No evidence is found for the suggestion that cross-border transport road infrastructure lags behind domestic transport infrastructure, as was suggested by Rietveld and Wintershoven (Rietveld and Wintershoven, 1998, Rietveld, 2001). We find that, in the study area, foreign highway-induced accessibility levels have increased more than domestic highway-induced accessibility in EU members than in the non-EU member. The role of the EU in inciting improvements in cross-border transport infrastructure certainly merits further study.

Domestic interaction opportunities provided by highway infrastructure have clearly affected population growth in the entire study area, confirming the expectation that market access matters for population growth (Brakman et al., 2012, Koopmans et al., 2012, Redding and Sturm, 2008). However, notwithstanding the considerable growth of cross-border accessibility in the study area, the population effects of cross-border transport infrastructure seem limited at best, indicating that in the study area local markets are not integrated across national borders. Through underuse of existing interaction opportunities, national borders continue to have a persistent negative effect on growth. In no case, accessibility levels yielded by cross-border transport supply have clearly positively affected the spatial distribution of population growth. The result that reduced access to proximate interaction opportunities causes that in particular border regions lag behind in terms of population growth confirms amongst others Brakman et al. (2012) and Redding and Sturm (2008). The enduring importance of national borders is further underlined in a recent analysis of house price differences on both sides of Dutch-German border (Micheli et al., 2014). If anything, the results here show that improving cross-border transport supply is not the solution to further integrate cross-border local markets.

We found that infrastructure supply is not lagging behind in border areas. Nevertheless, international economic integration has seemingly not affected the cross-border integration of local markets. The EU, arguably the most successful process of international economic integration in the world (McCormick, 1999), has removed many difficulties associated with crossing borders in the study area. However, according to the findings in this study, this has not affected Europe's patterns of growth, confirming that EU citizens remain to be relatively immobile compared to for example US citizens (Cheshire and Magrini, 2009). The barriers that borders impose will have to be explained from other, institutional factors such as complications in contract enforcement across borders (Rodrik, 2000) and cultural and linguistic differences (Chen, 2004, Linders, 2006, Persyn and Torfs, 2015).

Notwithstanding almost 60 years of international economic integration in a large part of the study area, the persistence of border effects is not completely surprising. Redding and Sturm (2008) have shown for the case of the division of Germany that, while introducing a border had substantial and almost immediate impacts on population growth, that growth responded less clearly to the formal removal of national borders after German reunification. In the case tackled by Redding and Sturm, the difficulties associated with national borders existed for a relatively small time, between culturally and linguistically homogeneous people. With the exception of Germany, the states studied in this paper exist more or less in their current form since the 19th century, and cultural and linguistic differences between these states further complicate the matter. All in all, it might take a long time until economic international integration is reflected in local growth patterns in the EU.

## Figures and Tables

**Table:** 

*B*_*i* , *t*_^*DOM*^ = ∑_*i* ≠ *j*_^*n*^(1 − *F*_*ij* , *t*_)*P*_*j*_(*M*_*ij* , *t*_^*base*^)^*γ*^	(A1.1)	Domestic base accessibility as a product of domestic populations and the modelled underlying network.
*T*_*i* , *t*_^*DOM*^ = *A*_*i* , *t*_^*DOM*^ − *B*_*i* , *t*_^*DOM*^	(A1.2)	Domestic highway-induced accessibility as a product of the observed highway network.
*B*_*i* , *t*_^*FOR*^ = ∑_*i* ≠ *j*_^*n*^*F*_*ij* , *t*_*P*_*j*_(*M*_*ij* , *t*_^*base*^)^*γ*^	(A1.3)	Foreign base accessibility spillovers as a product of cross-border populations and the modelled underlying network.
*T*_*i* , *t*_^*FOR*^ = *A*_*i* , *t*_^*FOR*^ − *B*_*i* , *t*_^*FOR*^	(A1.4)	Foreign highway-induced accessibility spillovers as a product of the observed highway network.

**Table 6:** Effect of municipal population densities per observed year per population density class.

Year	Pop. dens. (p./sq. km)	In EU since 1957	In EU since 1986	In EU since 1995	Not an EU member
1971	< 150⁎	− 0.37⁎⁎ (− 34.5)	− 0.36⁎⁎ (− 12.5)	− 0.28⁎⁎ (− 18.4)	− 0.66⁎⁎ (− 20.6)
	150–250	− 0.36⁎⁎ (− 47.2)	− 0.26⁎⁎ (− 11.4)	− 0.25⁎⁎ (− 20.5)	− 0.58⁎⁎ (− 25.3)
	250–500	− 0.32⁎⁎ (− 60.5)	− 0.17⁎⁎ (− 9.9)	− 0.25⁎⁎ (− 26.1)	− 0.51⁎⁎ (− 32.5)
	500–1000	− 0.31⁎⁎ (− 81.6)	− 0.24⁎⁎ (− 20.1)	− 0.27⁎⁎ (− 35.3)	− 0.45⁎⁎ (− 41.1)
	1000–2500	− 0.33⁎⁎ (− 126.8)	− 0.31⁎⁎ (− 32.9)	− 0.30⁎⁎ (− 43.7)	− 0.41⁎⁎ (− 47.7)
	2500–5000	− 0.35⁎⁎ (− 171.0)	− 0.25⁎⁎ (− 47.4)	0.45⁎⁎ (6.4)	− 0.41⁎⁎ (− 74.0)
	5000 >	− 0.44⁎⁎ (− 276.1)	− 0.31⁎⁎ (− 82.1)	0.30⁎⁎ (5.5)	− 0.45⁎⁎ (− 77.3)
1981	< 150	− 0.34⁎⁎ (− 31.5)	− 0.32⁎⁎ (− 11.4)	− 0.32⁎⁎ (− 22.2)	− 0.62⁎⁎ (− 19.7)
	150–250	− 0.34⁎⁎ (− 45.1)	− 0.22⁎⁎ (− 9.7)	− 0.30⁎⁎ (− 24.6)	− 0.59⁎⁎ (− 26.3)
	250–500	− 0.33⁎⁎ (− 64.1)	− 0.10⁎⁎ (− 6.8)	− 0.29⁎⁎ (− 32.5)	− 0.55⁎⁎ (− 37.5)
	500–1000	− 0.35⁎⁎ (− 94.9)	− 0.14⁎⁎ (− 12.9)	− 0.30⁎⁎ (− 38.5)	− 0.55⁎⁎ (− 56.3)
	1000–2500	− 0.40⁎⁎ (− 164.6)	− 0.16⁎⁎ (− 21.0)	− 0.35⁎⁎ (− 55.2)	− 0.53⁎⁎ (− 70.8)
	2500–5000	− 0.44⁎⁎ (− 224.2)	− 0.31⁎⁎ (− 60.8)	0.40⁎⁎ (5.7)	− 0.53⁎⁎ (− 93.8)
	5000 >	− 0.49⁎⁎ (− 290.0)	− 0.36⁎⁎ (− 108.8)	0.39⁎⁎ (6.2)	− 0.59⁎⁎ (− 100.2)
1991	< 150	− 0.40⁎⁎ (− 38.1)	− 0.27⁎⁎ (− 9.8)	− 0.30⁎⁎ (− 20.5)	− 0.47⁎⁎ (− 14.7)
	150–250	− 0.39⁎⁎ (− 53.3)	− 0.20⁎⁎ (− 9.3)	− 0.28⁎⁎ (− 23.5)	− 0.43⁎⁎ (− 19.0)
	250–500	− 0.39⁎⁎ (− 77.5)	− 0.15⁎⁎ (− 10.2)	− 0.29⁎⁎ (− 32.6)	− 0.45⁎⁎ (− 30.6)
	500–1000	− 0.39⁎⁎ (− 108.6)	− 0.17⁎⁎ (− 16.9)	− 0.30⁎⁎ (− 40.6)	− 0.48⁎⁎ (− 49.6)
	1000–2500	− 0.42⁎⁎ (− 175.7)	− 0.27⁎⁎ (− 43.7)	− 0.34⁎⁎ (− 54.7)	− 0.50⁎⁎ (− 65.4)
	2500–5000	− 0.47⁎⁎ (− 229.0)	− 0.33⁎⁎ (− 71.8)	0.48⁎⁎ (6.5)	− 0.50⁎⁎ (− 79.8)
	5000 >	− 0.54⁎⁎ (− 296.7)	− 0.44⁎⁎ (− 139.4)	0.43⁎⁎ (6.2)	− 0.50⁎⁎ (− 75.4)
2001	< 150	− 0.38⁎⁎ (− 36.9)	− 0.30⁎⁎ (− 10.8)	− 0.28⁎⁎ (− 20.3)	− 0.50⁎⁎ (− 16.7)
	150–250	− 0.37⁎⁎ (− 51.3)	− 0.22⁎⁎ (− 11.0)	− 0.26⁎⁎ (− 23.0)	− 0.46⁎⁎ (− 21.1)
	250–500	− 0.37⁎⁎ (− 75.6)	− 0.17⁎⁎ (− 13.0)	− 0.28⁎⁎ (− 31.3)	− 0.45⁎⁎ (− 32.7)
	500–1000	− 0.38⁎⁎ (− 106.8)	− 0.18⁎⁎ (− 18.8)	− 0.29⁎⁎ (− 40.0)	− 0.48⁎⁎ (− 51.7)
	1000–2500	− 0.42⁎⁎ (− 174.3)	− 0.30⁎⁎ (− 49.9)	− 0.39⁎⁎ (− 62.0)	− 0.50⁎⁎ (− 69.1)
	2500–5000	− 0.45⁎⁎ (− 215.7)	− 0.40⁎⁎ (− 93.6)	0.49⁎⁎ (6.6)	− 0.51⁎⁎ (− 79.7)
	5000 >	− 0.53⁎⁎ (− 272.0)	− 0.51⁎⁎ (− 154.1)		− 0.52⁎⁎ (− 80.5)
2011	< 150	− 0.36⁎⁎ (− 36.2)	− 0.22⁎⁎ (− 8.4)	− 0.29⁎⁎ (− 22.2)	− 0.50⁎⁎ (− 17.5)
	150–250	− 0.36⁎⁎ (− 52.0)	− 0.14⁎⁎ (− 7.0)	− 0.27⁎⁎ (− 25.0)	− 0.45⁎⁎ (− 21.8)
	250–500	− 0.36⁎⁎ (− 76.3)	− 0.10⁎⁎ (− 7.5)	− 0.26⁎⁎ (− 30.0)	− 0.41⁎⁎ (− 30.9)
	500–1000	− 0.36⁎⁎ (− 105.7)	− 0.11⁎⁎ (− 12.1)	− 0.27⁎⁎ (− 38.1)	− 0.41⁎⁎ (− 45.8)
	1000–2500	− 0.40⁎⁎ (− 166.3)	− 0.21⁎⁎ (− 36.4)	− 0.30⁎⁎ (− 47.0)	− 0.44⁎⁎ (− 63.0)
	2500–5000	− 0.43⁎⁎ (− 204.0)	− 0.35⁎⁎ (− 94.9)	0.58⁎⁎ (7.9)	− 0.47⁎⁎ (− 72.0)
	5000 >	− 0.45⁎⁎ (− 230.7)	− 0.44⁎⁎ (− 119.7)		− 0.47⁎⁎ (− 73.8)

Population density (pop. dens.) classes are expressed in people per square kilometer.

⁎ p < 0.05.

⁎⁎ p < 0.01.

**Fig. 4:**
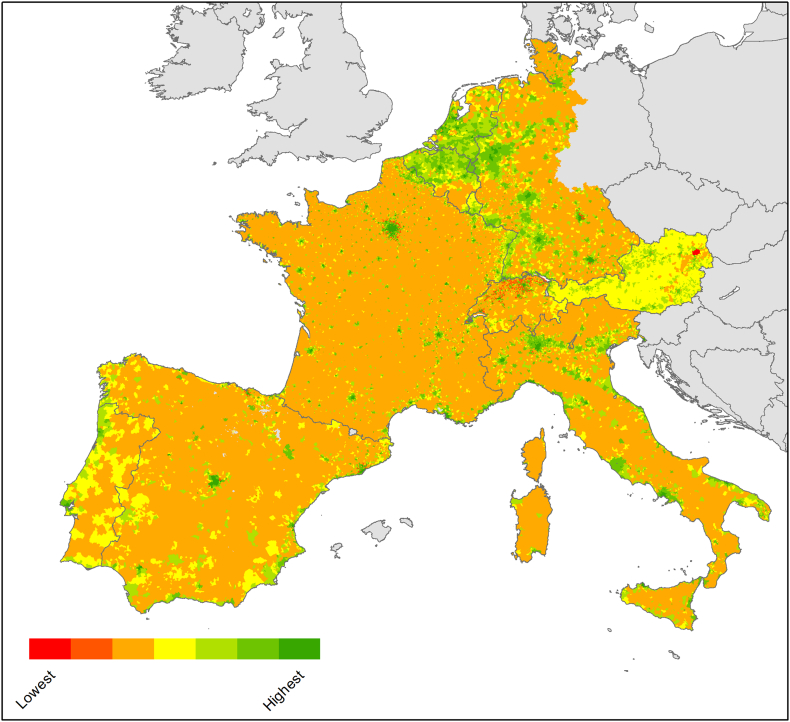
Municipal fixed effects estimated in the model presented in Section 4.

## Appendix I Definition of all used accessibility variables

This appendix presents the various components of accessibility that are introduced in Section 2.

## Appendix II Additional results population growth model

This appendix shows the additional results from the model presented in Section 4.

## Footnotes

2 Results are available upon request.

3 Results are available upon request.

4 Results are available upon request.
